# Objective Imaging Diagnostics for Dry Eye Disease

**DOI:** 10.1155/2020/3509064

**Published:** 2020-07-22

**Authors:** Sang Beom Han, Yu-Chi Liu, Karim Mohamed-Noriega, Louis Tong, Jodhbir S. Mehta

**Affiliations:** ^1^Department of Ophthalmology, Kangwon National University School of Medicine, Kangwon National University Hospital, Chuncheon, Republic of Korea; ^2^Singapore National Eye Centre, Singapore; ^3^Singapore Eye Research Institute, Singapore; ^4^Department of Ophthalmology, Yong Loo Lin School of Medicine, National University of Singapore, Singapore; ^5^Department of Ophthalmology, University Hospital, Faculty of Medicine, Autonomous University of Nuevo Leon, Monterrey, Mexico

## Abstract

Traditional diagnostic tests for dry eye disease (DED), such as fluorescein tear film break-up time and the Schirmer test, are often associated with poor reproducibility and reliability, which make the diagnosis, follow-up, and management of the disease challenging. Advances in ocular imaging technology enables objective and reproducible measurement of changes in the ocular surface, tear film, and optical quality associated with DED. In this review, the authors will discuss the application of various imaging techniques, such as, noninvasive tear break-up time, anterior segment optical coherence tomography, in vivo confocal microscopy, meibography, interferometry, aberrometry, thermometry, and tear film imager in DED. Many studies have shown these devices to correlate with clinical symptoms and signs of DED, suggesting the potential of these imaging modalities as alternative tests for diagnosis and monitoring of the condition.

## 1. Introduction

Dry eye disease (DED) is a multifactorial disease characterized by a loss of homeostasis of the tear film [1]. It a highly prevalent disease that can affect up to one-third of the people worldwide [2, 3]. The disease is associated with symptoms such as ocular discomfort, dryness, pain, foreign body sensation, and visual disturbance [4, 5], which significantly interfere with daily activities including reading, driving, watching TV, and using mobile devices or computer [2, 3]. In clinical practice, the diagnosis and management of DED is often difficult because of its multifactorial nature, as well as discrepancy between dry eye signs and symptoms [2, 6–8]. Moreover, conventional diagnostic tests for DED, such as the fluorescein tear film break-up time (TBUT) and the Schirmer test, often show unsatisfactory reliability and reproducibility, which also renders the diagnosis and monitoring of the condition challenging [9].

Advances in technology led to the development of various imaging devices that have enabled visualization and evaluation of the tear film and ocular surface, such as noninvasive tear break-up time measurements, anterior segment optical coherence tomography, confocal microscopy, meibography, interferometry, aberrometry, thermography, and tear film imager [10].

In this review, we aim to provide an overview of imaging devices for DED and discuss the application of the modalities for clinical practice and research for DED.

## 2. Noninvasive Tear Break-Up Time (NITBUT)

The TBUT reflects the stability and quality of the tear film, which is crucial for maintenance of ocular surface integrity and clear vision [1, 11]. Measurement of the TBUT has generally been performed after fluorescein dye instillation [10]. However, the variability of the concentration and amount of the dye leads to reduced reliability and reproducibility [12]. Reflex tearing induced by fluorescein instillation can also lead to decreased accuracy [12]. Moreover, the fluorescein TBUT is unable to simultaneously evaluate the tear break-up across the entire corneal surface [10].

**Figure 1:**
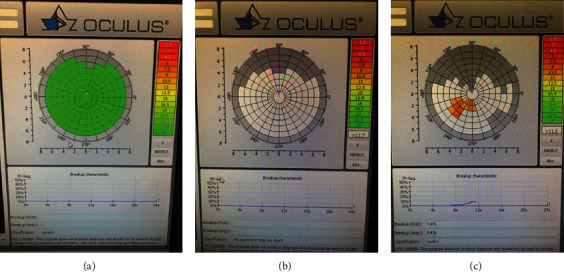
Noninvasive tear film break-up time (NIBUT) using the Oculus Keratograph 5 M (Oculus Optikgeräte GmbH, Wetzlar, Germany) presented as a tear film break-up color-code map. (a) No tear film break-up by 22–23 sec. (b) Immediately after tear film break-up. (c) At 7 sec after blinking.

The noninvasive TBUT (NIBUT) was developed to overcome these limitations [10, 11]. Instead of using fluorescein dye instillation, the NIBUT measurement involves application of topographic systems to evaluate changes in a regularly patterned image projected onto the tear film that reflects compromised tear film integrity [11, 13]. Changes in reflected videokeratographic mires or grids from an illuminated placido disc are observed to detect tear film disruption (Figure 1) [13].

The NIBUT was shown to have a correlation with the dry eye symptom score and a good diagnostic value for DED [14, 15]. Dry Eye Workshop II (DEWS II) suggested the NIBUT with a cutoff value of ≤10 seconds as an indicator for diagnosis of DED with 82% to 84% sensitivity and 76% to 94% specificity [16]. The NIBUT was also revealed to be useful for monitoring of treatment response in DED [15].

Bandlitz et al. [17] recently reported that objective measurement of the NIBUT using the Keratograph 5 M (Oculus Optikgeräte GmbH, Wetzlar, Germany) showed good repeatability and reasonable agreement with subjective NIBUT measurement using the Tearscope Plus (Keeler, Windsor, UK), Polaris (bon Optic, Lübeck, Germany), and EasyTear Viewplus (Easytear, Rovereto, Italy), which also support viability of the NIBUT in the diagnosis and treatment of DED [15].

However, measurement of the NIBUT using two different topography platforms, the Keratograph 5 M and RT-7000 Auto Refractor-Keratometer (Tomey, Nagoya, Japan), showed poor agreement [18], suggesting that measured values using different topographers with different algorithms should not be assessed interchangeably [10].

Although the NIBUT has a correlation with the fluorescein TBUT, its results should be carefully interpreted as it actually evaluates the thinning of the tear film, not the break-up of the full-thickness of the tear film [19].

## 3. Anterior Segment Optical Coherence Tomography

Anterior segment optical coherence tomography (AS-OCT) produces cross-sectional images of anterior segment structures by low-coherence interferometry [10, 20]. The technique enables measurement of tear meniscus parameters important for the diagnosis and monitoring of DED [16, 21–26], such as the tear meniscus height (TMH) and tear meniscus area (TMA) [10, 20, 27], without reflex tearing due to its noncontact nature and rapid image acquisition [11, 27].

Ibrahim et al. [22] showed that the TMH measured by time-domain (TD) OCT was correlated with strip meniscometry, corneal staining scores, and the Schirmer score. Spectral-domain (SD) OCT enabled higher resolution and faster image acquisition compared to TD-OCT, resulting in improved image quality with minimal artifact, as well as enhanced repeatability [27–29]. SD-OCT allowed improved sensitivity and specificity for the TMH and TMA as diagnostic biomarkers for DED compared to TD-OCT [11, 22]. SD-OCT findings also showed a close correlation with dry eye symptoms and the Schirmer score [24]. Qiu et al. [30] reported that diagnostic accuracy of SD-OCT was the highest for Sjögren's syndrome, moderately acceptable for non-Sjögren's aqueous-deficient DED, and the lowest for evaporative DED, suggesting the SD-OCT can be a viable option in the diagnosis of aqueous-deficient DED [30]. AS-OCT was also shown to be able to accurately measure the thickness of the overall tear film [31, 32]. Sher et al. [33] demonstrated that AS-OCT may also be helpful for the quantitative evaluation of corneal epithelial erosion.

AS-OCT is also useful for monitoring of treatment responses in DED [34–36]. Measurement of the TMH using TD-OCT might be effective in monitoring tear meniscus changes after punctal occlusion [35]. SD-OCT was useful for quantifying the sequential changes of tear meniscus parameters after artificial tear instillation [37]. Nagahara et al. [38] showed that the TMH decreased with CL wear and increased after the instillation of diquafosol sodium. Although the TMH and TMA measured using AS-OCT may be valuable biomarkers for DED [11], factors including the time-from-blink, palpebral aperture, presence of conjunctivochalasis, and lid length should be considered in the interpretation of these tear meniscus measurements [39].

Swept source OCT (SS-OCT) enables acquisition of three-dimensional images, as well as an enhanced scanning speed and greater imaging depth compared to TD and SD-OCT [40], which allows the measurement of the tear meniscus volume (TMV) in addition to the TMH and TMA [41]. The TMH, TMV, and TMA measured using SS-OCT showed a correlation with the corneal staining score, BUT, and Schirmer score [25]. All three parameters showed the strongest correlation with the Schirmer score, suggesting that the tear meniscus parameters mostly reflect the quantity of tear fluid [25]. SS-OCT was also useful for evaluation of increased tear meniscus parameters after installation of eye drops, such as sodium hyaluronate, diquafosol, and rebamipide [34].

En-face OCT may be useful for observation of changes in the ocular surface, particularly due to its noncontact nature and large scan width [42]. Ghouali et al. [43] evaluated the palisade of Vogt using this device to determine the changes in the limbal anatomy in DED patients and showed that the visibility score of the palisades was lower in DED patients with a decreased score in accordance with the severity of DED [43].

AS-OCT is expected to be useful for the diagnosis of meibomian gland dysfunction (MGD), one of the most common causes of DED [44]. Hwang et al. [45] introduced a method of developing 3D images of meibomian glands (MGs) by reconstructing a series of tomograms of MGs captured by high-speed SD-OCT. SS-OCT can provide 3D high-resolution images of MG acini and ducts that cannot be observed by infrared meibography [46]. OCT meibography showed a decreased MG length and width in obstructive MGD, which correlated with ocular surface symptoms and signs [47]. However, so far, there are only preliminary data; thus, further studies are needed for clinical application of MG imaging using OCT [48].

## 4. In Vivo Confocal Microscopy

In vivo confocal microscopy (IVCM) a noninvasive method that provides real-time imaging of the ocular surface at the histologic level, which enables the evaluation of changes in cells reflecting ocular surface damage and inflammation, such as corneal epithelial cells, keratocytes, and dendritic cells [1, 10, 49, 50]. IVCM also allows for observation of changes in the corneal nerve associated with DED [49].

In 2003, Tuominen et al. [51] reported that IVCM demonstrated reduced central corneal thickness, patchy alterations or irregularities in corneal epithelial cells, activated keratocytes reflected by abnormal hyper-reflectivity, and abnormal morphology of sub-basal nerve fiber bundles resembling nerve sprouting, suggesting active neural regeneration in Sjögren's syndrome [51]. Villani et al. [52] showed that IVCM revealed reduced density of the superficial epithelial cells, as well as decreased central corneal thickness in Sjögren's syndrome [52]. Other studies also showed decreased cell densities in the superficial corneal epithelial layer in both Sjögren's syndrome and non- Sjögren's DED [53–55].

Several studies showed decreased sub-basal nerve density, as well as increased number of beadings and nerve tortuosity in both Sjögren's syndrome and non-Sjögren's DED [50, 52, 54–57]. These changes in corneal nerves had correlation with corneal sensitivity, dry eye symptoms, and the Schirmer score [55–57]. By contrast, Zhang et al. [58] reported that IVCM showed increased corneal nerve density in Sjögren's syndrome. However, they also reported increased tortuosity in corneal nerves in Sjögren's syndrome [58]. Morphologic changes including beading and tortuosity may reflect an attempted regeneration of the corneal nerve [51, 52, 55] and suggested to be indices of metabolic activity of the sub-basal nerve plexus [57].

**Figure 2:**
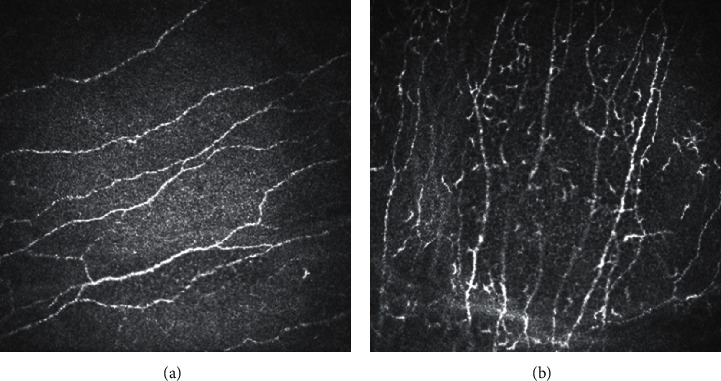
In vivo confocal microscopy. (a) Normal sub-basal nerve plexus. (b) Increased dendritic cells in dry eye disease.

Dendritic cells are antigen-presenting cells that play an important role in ocular surface immunology [59]. In DED, dessication stress causes proinflammatory stimulation on the ocular surface, which conceivably promotes migration and maturation of dendritic cells (Figure 2) [59]. Lin et al. [60] demonstrated increased dendritic cells in the central cornea in both Sjögren's syndrome and non-Sjögren's DED [60]. They also revealed an increased number of “activated” dendritic cells characterized by the increased dendrites [60]. Kheirkhah et al. [61] demonstrated marked increase in dendritic cell density at the sub-basal epithelial region in aqueous-deficient immunologic DED, such as Sjögren's syndrome and graft versus host disease (GVHD), compared with the aqueous-deficient nonimmunologic DED, evaporative DED, and controls [61].

IVCM was shown to be useful for the monitoring of treatment responses in DED [59]. Using an in vivo laser-scanning confocal microscope (Heidelberg Retina Tomograph, Rostock Corneal Module (HRT-RCM); Heidelberg Engineering GmgH, Heidelberg, Germany), Villani et al. [62] demonstrated that the density of sub-basal dendritic cells and activated keratocytes significantly decreased after 4 weeks of treatment with topical steroid. Iaccheri et al. [63] reported increased cell density of the corneal intermediate epithelium, decreased activated keratocytes, and reduced tortuosity of corneal nerve fibers after 6 months of treatment with 0.05% topical cyclosporine in DED [63]. Levy et al. [64] also reported an increase in corneal sub-basal nerves and a decrease in dendritic cell density after 6 months of treatment with topical 0.05% cyclosporine in Sjögren's syndrome. IVCM also demonstrated decreased corneal basal epithelial cell density and reduced numbers of nerve beadings after treatment with autologous serum eye drops [65, 66].

**Figure 3:**
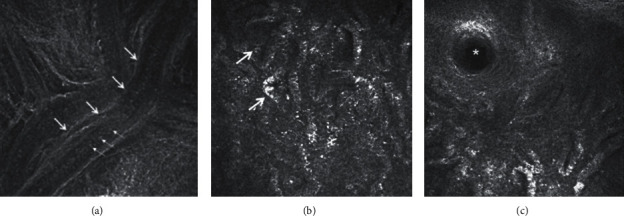
(a) In vivo confocal microscopy showing the meibomian gland duct of one of the meibomian glands of the upper eyelid. Large arrows show the wall of the terminal duct and small arrows show the meibum within. (b) The multiple irregular globular structures (arrows) indicate the acini of meibomian glands. (c) The orifice of a single meibomian gland is shown by the asterix.

IVCM can also provide high-resolution imaging of the MGs; thus, it can be useful for the evaluation of the MG morphology at a cellular level, which is important for the diagnosis of MGD (Figure 3) [10, 67]. IVCM enabled determination of novel MG parameters, e.g., meibomian gland acinar unit density (MGAUD), shortest diameter (MGASD) and longest diameter (MGALD), and inflammatory cell density [67, 68]. These parameters have a significant correlation with tear film parameters, ocular surface signs, and MG expressibility [67, 68].

IVCM with HRT-RCM demonstrated morphologic alterations in MGD, such as enlargement of glandular acinar units, extensive periglandular inflammatory cell infiltration, and hyperkeratinization of the ductal epithelium [67–69]. In severe MGD, atrophy in MGs with extensive periglandular fibrosis was observed [70].

Ibrahim et al. [68] revealed that MGD was associated with lower MGAUD, larger MGASD and MGALD, and higher inflammatory cell density. Matsumoto et al. [70] also reported similar findings and showed the acinar unit density and diameters had association with the severity of MG dropout and expressibility. They also showed a significant reduction in the inflammatory cell density of MGs after treatment with lid hygiene, topical 0.5% levofloxacin and 0.1% fluorometholone, and oral 100 mg minocycline [70]. Ban et al. [71] demonstrated that patients with DED/GVHD had significantly lower MGAUD, shorter MGASD and MGALD, and a higher fibrosis grade compared to those with non-DED/non-GVHD. Villani et al. [72] revealed that patients with Sjögren's syndrome had greater acinar density, shorter diameters, higher density of periglandular inflammatory cells, and lower secretion reflectivity compared with those with MGD.

However, IVCM has a limitation that it is not capable of cellular and tissue phenotyping, and analysis is only based on morphology and reflectivity [48]. The small field of view (<0.25 mm [2]) is also a major limitation of the device [48].

## 5. Infrared Meibography

**Figure 4:**
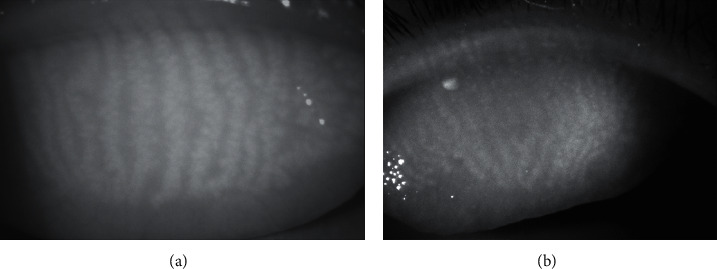
(a) Infrared meibography showing relatively normal meibomian glands in the upper eyelid. Brighter areas indicate glandular areas, whereas darker areas indicate intergland tissue. (b) Meibography showing slight atrophy of the meibomian glands in the proximal margin of the tarsal plate. The abnormal area is greyish without typical whitish tracks that represent the glands.

Noncontact infrared meibography based on combined transillumination with infrared photography provides two-dimensional silhouette of MGs [10, 73]. The technique has been widely used for the evaluation of MG dropout since its introduction in 2008, as it can provide improved image quality with a short acquisition time and minimal patient discomfort (Figure 4) [74]. In meibography, healthy meibum is visualized as a light area due to its autofluorescence [11, 73]. Dark areas in the MG conceivably indicate loss of acinar tissue or an altered meibum condition, which is determined as MG dropout [73, 75].

Among various grading scales proposed for MG dropout [76], the Gestalt grading scale and meiboscale were recommended by the MGD Workshop [74, 75]. In the Gestalt grading scale, grading of each lid is performed on a scale from 1 to 4, based on the ratio of partial glands as follows [11]: grade 1 = no partial glands; grade 2 = less than 25% partial glands; grade 3 = 25% to 75% partial glands; and grade 4 = greater than 75% partial glands [11]. In the meiboscale, each lid is graded based on the ratio of MG dropout: grade 0 = no loss of MGs; grade 1 = area loss ≤ 25%; grade 2 = area loss ≤ 50%; grade 3 = area loss ≤ 75%; and grade 4: area loss ≤ 100% [77].

Recently, continuous grading scales using semiautomated software that automatically calculates the ratio of the area of MG loss to the total area of the eyelid have been developed [11, 78]. As the ratio of MG dropout is expressed as numeric values ranging from 0 to 100, the continuous scales may be advantageous for evaluating subtle changes that may not be detected using categorical grading scales and can facilitate the determination of efficacy of treatment, such as intraductal probing and eyelid warming [11, 79, 80].

The area of MG dropout showed a positive correlation with the meibum grade [81, 82]. Both the severity of MGD and the percentage of MG dropout had a correlation with the TBUT, dry eye symptom score, and corneal stain score [83]. The ductal length and acini area measured by meibography were correlated with the tear film, corneal stain score, and meibum level [84]. Arita et al. [85] demonstrated the correlation between MG dropout and Schirmer's score in MGD, suggesting that tear fluid production may increase to compensate for tear film instability due to MGD. MG dropout measured using infrared meibography has proven to be useful for differential diagnosis of MGD and aqueous deficiency DED [86].

However, infrared meibography has a limitation that it cannot provide three-dimensional images of the deeper structures [10]. Hence, the MG dropout evaluated by infrared meibography should be carefully interpreted [10]. Additional information using AS-OCT or IVCM might be helpful for the diagnosis and monitoring of MGD.

## 6. Interferometry

Tear interferometry is a noninvasive method for the investigation of the tear lipid layer by visualization of the reflection of light at the lipid-aqueous interface of the tear film [10, 87, 88]. Interferometry allows objective evaluation of tear film properties, such as lipid layer thickness, break-up characteristics and changes in thickness of the tear film, its distribution, and wetting patterns with sequential blinking [81, 89, 90].

Yokoi et al. [91] showed that grading of the lipid layer interference pattern had a significant correlation with the corneal staining score and TBUT. Goto et al. [92] developed a tear interference color chart for the DR-1 *α* interferometer (Kowa, Nagoya, Japan), which can be useful for converting tear interference color information to the lipid layer thickness (LLT) [92]. Subsequently, Arita et al. [93] showed that the DR-1*α* interferometer could measure the TMH as reliably as SS-OCT and showed that the interferometric TMH had correlation with Schirmer's score. Arita et al. [88] also described a difference in interferometric patterns among aqueous-deficient DED, MGD, and normal control on the DR-1*α* interferometry, suggesting that the device can be helpful for differential diagnosis of subtypes of DED.

The DR-1*α* interferometer is also capable of kinetic analysis of spread and stability of the lipid layer [94]. MGD was associated with significantly increased lipid spread time [94]. The kinetic analysis can also be helpful for evaluation of the improvement of the lipid layer after treatment, e.g., low-dose lipid application on the lid margin or punctal occlusion [95, 96].

The LipiView II (LVII) ocular surface interferometer (TearScience, Johnson and Johnson Vision, Jacksonville, FL, USA) is capable of providing quantitative information of the LLT, as well as images of MGs using a patented Lid Everter and infrared diodes for eversion and illumination of the eyelid [10, 97]. Ji et al. [81] showed that LLT measured using the LVII had a significant correlation with the TBUT, MGD grade, and MG dropout, suggesting that it can be an alternative to traditional dry eye tests. Eom et al. [98] also revealed that LLT measured with LVII was negatively correlated to MG loss and obstructive MGD was associated with lower LLT [98]. LLT may reflect the changes in meibum secretion and be helpful for differential diagnosis of MGD [10]; LLT would be increased in hypersecrectory MGD and decreased in obstructive MGD [99]. However, the LLT should be carefully interpreted as it can be affected by factors, such as age, sex, and ocular surgical history [100].

## 7. Wavefront and Double-Pass Aberrometry

DED is often associated with complaints including blurred vision, glare, and fluctuating vision with blinking, which is difficult to measure by conventional visual acuity testing [19, 101]. As the air-tear film interface forms the first refractive component of the eye, irregularity of the tear film in DED may cause decreased optical quality [10].

Tear film instability increases higher-order aberrations (HOAs) and ocular forward light scattering, resulting in “fluctuating vision with blinking” and “glare,” respectively [101]. Damage in the central cornea, i.e., the overlying optical zone, is associated with increased HOAs and corneal backward light scattering, leading to “blurred vision” [101].

Quantification of HOA using wavefront sensors, such as the Shack–Hartmann aberrometer, allows evaluation of optical aberrations associated with DED [102–104]. Wavefront sensing has shown that break-up of the tear film was associated with increased HOAs both in photopic and scotopic conditions [102]. Sequential measurement of HOAs demonstrated that superficial punctate keratopathy may aggravate both baseline HOAs and sequential postblink changes in HOAs in patients with DED [105]. Sequential wavefront measurements in eyes with a short TBUT have shown that a prolonged blink interval leads to increased HOAs with a marked upward curve after blinking, suggesting that suppressed blinking, such as while working with a video display terminal, can result in reduced optical quality [106]. Denoyer et al. [107] also reported a progressive increase in postblink HOAs in patients with DED and the correlation of the changes in HOAs with the OSDI score and TBUT [107].

Evaluation of the objective scatter index (OSI) obtained using the double-pass image of a point source projected on the retina enables the quantification of the ocular light scattering that cannot be measured using conventional wavefront sensors [104, 108, 109]. Using the double-pass aberrometer, Tan et al. [110] showed an increase in the OSI in patients with DED and the correlation between the OSI change and severity of DED. The rate of change in the OSI was correlated to the corneal staining score [111]. Koh et al. [112] revealed that DED was associated with greater ocular forward light scattering and corneal backward light scattering. Superficial punctate keratopathy overlying the optical zone was related to increased corneal backward light scattering [112].

An improvement in HOA and light scattering was reported after instilling artificial tear drops in patients with DED [113]. Serial examinations showed increased HOAs and forward light scattering immediately after the instillation of a highly viscous 0.3% sodium hyaluronate solution and the instillation of 2% rebamipide suspension, respectively, accounting for a temporal reduction in optical quality after instillation of eye drops with high viscosity or suspensibility [114].

## 8. Thermography

Infrared thermography is used to measure the amount of infrared radiation emitted from the ocular surface using an infrared thermal camera; thus, it allows a noninvasive evaluation of the changes in the ocular surface temperature (OST) caused by tear fluid evaporation [10, 115]. Decrease in the OST had a correlation with the TBUT and NIBUT [116, 117], conceivably, because tear film instability facilitates tear fluid evaporation and heat loss [116]. Su et al. [115] demonstrated that the OST difference 3 seconds after blinking was correlated with the TMH and Schirmer score.

Using a thermographic device (Ocular Surface Thermographer; OST, Tomey, Nagoya, Japan), Kamao et al. [117] reported that DED was associated with a greater decrease in the OST at 10 seconds after eye opening and suggested that changes in the OST could be an indicator for DED. Tan et al. [118] also showed that the temperature of the extreme nasal conjunctiva at 5 and 10 seconds after eye opening was a good detector for DED. Arita et al. [119] revealed that obstructive MGD was associated with lower surface temperature of the tarsal conjunctiva, which might increase the viscosity of the meibum and result in obstruction of the glands [119].

## 9. Tear Film Imager

The tear film imager (TFI) is a new technology that provides real-time images of the mucoaqueous and lipid tear layers [120]. Using spectral interference technology, this instrument enables noninvasive measurement of parameters including the thickness of mucoaqueous and lipid layers, thickness change rate, and the break-up time with a large field of view and nanometer axial resolution [120, 121].

Segev et al. [121] recently revealed that the device can reproducibly measure the mucoaqueous thickness which correlates with the Schirmer score. Lipid break-up time measurement with the TFI had a correlation with the TBUT [121]. DED was associated with lower mucoaqueous thickness and shorter lipid break-up time [121]. Cohen et al. [122] demonstrated that the TFI was capable of creating detailed maps of the lipid layer thickness and quantifying the lipid map uniformity due to its nanometer thickness resolution, which could be helpful for the diagnosis and treatment of DED.

## 10. Conclusions

Advances in ocular imaging technology have enabled objective and reproducible evaluation of ocular surface change, tear film parameters, and optical quality associated with DED; thus, they can be useful for diagnosis and management of the disease, as well as elucidation of its pathogenesis [123].

Studies have indicated the efficacy of various imaging modalities, i.e., the NIBUT for evaluation of the TBUT, AS-OCT for quantification of tear meniscus parameters, IVCM for high-resolution visualization of the ocular surface, infrared meibography for evaluation of MG dropout, interferometry for the measurement of the tear lipid layer, wavefront aberrometry and the OSI for the quantification of optical quality, thermography for the detection of changes in the OST, and the TFI for the evaluation of mucoaqueous and lipid layers [10].

With technological developments, these imaging devices are expected to provide more precise and accurate information on structural and functional changes associated with DED. Further studies are, therefore, warranted for clinical application of these devices and establishment of guidelines for the use of the modalities for diagnosis and management of DED.
